# The role of nicotine replacement therapy for temporary abstinence in the home to protect children from environmental tobacco smoke exposure: a qualitative study with disadvantaged smokers

**DOI:** 10.1186/1471-2458-13-262

**Published:** 2013-03-22

**Authors:** Olesya Atkinson, Tim Coleman, Ann McNeill, Sarah Lewis, Laura L Jones

**Affiliations:** 1 UKCTCS and Division of Primary Care, University of Nottingham, Nottingham, UK; 2 UKCTCS and National Addiction Centre, Institute of Psychiatry, King's College London, London, UK; 3 UKCTCS and Division of Epidemiology and Public Health, University of Nottingham, Nottingham, UK

**Keywords:** Environmental tobacco smoke, Nicotine replacement therapy, Temporary abstinence, Smoke-free home, Qualitative, Interview, Caregivers

## Abstract

**Background:**

Nicotine replacement therapy (NRT) has recently been licensed to help smokers to abstain from smoking for short time periods and recent studies have shown that 8-14% of smokers are regularly using NRT to cope when they cannot or are not allowed to smoke. These data suggest that, potentially, NRT for temporary abstinence might be an acceptable method to help smoking caregivers, who are not able to stop smoking completely, to avoid smoking whilst inside their home in order to protect their children from the harms of environmental tobacco smoke (ETS). The aim of this study was therefore to explore the concept of using NRT for temporary abstinence in the home, to protect children from exposure to ETS.

**Methods:**

Qualitative in-depth interviews were conducted with thirty six disadvantaged smoking parents who were currently, or had recently stopped smoking in the home with at least one child under the age of five. Parents were recruited from Children’s Centres and Health Visitor Clinics in Nottingham, UK. Interviews were audio recorded and transcribed verbatim. Data were coded and analysed thematically to identify emergent main and subthemes.

**Results:**

Overall, participants responded negatively to the concept of attempting temporary abstinence in the home in general and more specifically to the use of NRT whilst at home to reduce children’s exposure to ETS. Many parents would prefer to either attempt cutting down or quitting completely to make a substantial effort to change their smoking behaviour. There was limited interest in the use of NRT for temporary abstinence in the home as a first step to quitting, although some parents did express a willingness to use NRT to cut down as a first step to quitting.

**Conclusion:**

Disadvantaged smoking parents were reluctant to initiate and maintain temporary abstinence with or without NRT as a way of making their homes smoke free to protect their children’s health. More education about the specific risks of ETS to their children and the utility of NRT for use in the home might be needed to have a public health impact on children’s health.

## Background

Exposure to environmental tobacco smoke (ETS) in children is a major public health problem, causing serious health consequences [1-3]. Around two million children in the UK are regularly exposed to ETS, with the home being the main source of exposure. Children’s exposure to ETS is mainly determined by parental smoking [4]. The magnitude of the problem was recognised in the recent Department of Health’s tobacco control strategy for England, which aimed to increase the proportion of households where parents abstain from smoking inside the home from 21% in 2007 [4] to 66% by 2020 [5]. The most reliable way to protect children from ETS in the home is to get parents to quit smoking altogether. If quitting is not possible, then a complete smoking ban in the home is the next best option, as partial restrictions, such as smoking in one room only, are not effective at reducing exposure [6].

Nicotine replacement therapy (NRT) is an effective and widely used treatment for smoking cessation [7,8]. Some types of NRT are now also licensed for temporary abstinence, that is, to help smokers to abstain from smoking for short time periods [9]. A recent cross-sectional monthly survey of over 11 400 socio-economically diverse English smokers found that 14% regularly used NRT for temporary abstinence in situations when they were not allowed to smoke [10]. This study did not specifically differentiate between enforced temporary abstinence such as due to restrictions in the workplace and optional temporary abstinence, such as in private homes. However, this suggests that NRT used for optional temporary abstinence might be an acceptable method to help smokers, who are not able to stop smoking completely, to avoid smoking whilst inside their homes. As far as the authors are aware there have been no studies which have specifically investigated the use of NRT for temporary abstinence in this particular context. Consequently, this study aimed to determine smokers’ views about using NRT to help them abstain from smoking in their homes in order to protect their children’s health. We specifically sought the views of socially-economically disadvantaged families with children aged under five years because tobacco smoke exposure is highest amongst disadvantaged children [11] and younger children suffer the greatest morbidity from ETS exposure [1].

## Methods

### Design

Two sets of qualitative in-depth interview data (n=36) were combined in the current study to explore the views of smokers with young children on using NRT to abstain from smoking in the home. One-to-one interviews were chosen in both studies to enable open discussion of anticipated sensitive social and psychological issues [12-14]. In addition, this allowed the interviewers to offer flexible appointment slots to fit around the participants’ busy family lives. In the first study, 22 smoking families were interviewed between July and September 2009. As described previously [15], recruitment was done via Sure Start Children’s Centres which are located amongst the most socio-economically disadvantaged parts of Nottingham as defined by Index of Multiple Deprivation, and where the smoking rate was up to 48.1% in recent years [16]. In the second study, 14 smoking families were interviewed between October and November 2010. Potential participants were identified through six health visitor clinics, which were randomly selected from the 17 clinics located in areas across Nottingham City where we had not attempted to recruit families for the first study. The inclusion criteria for the participants were: age 16 years and over, self-reported current smoker and currently or recently smoking in the home, caregiver to at least one child under the age of five, and good level of spoken English. We purposively included parents who reported no longer smoking in the home. This was partly because other studies suggest that smoking rules are fluid and therefore self-report of having a smoke-free home may not be valid [12,14,17]. Secondly, parents who indeed did manage to establish smoke-free homes could provide invaluable insight into how to successfully implement changes of smoking behaviour in the home. The interviews took place in a private room at the Children’s Centre or the health visitor clinic where the participant accessed services or in the participant’s own home. All participants were offered a £15 retail voucher as an inconvenience allowance and for potentially having to arrange child care. At the end of the interview each participant completed a brief demographic and smoking information questionnaire.

These two interview studies form part of a larger body of research which aims to develop an effective intervention to reduce children’s exposure to domestic ETS. The aim of the first study was to explore home smoking behaviours, the motivators and barriers to smoke-free homes, and to identify the positive levers that health care professionals can utilise when supporting smoking caregivers to change their behaviours. The aim of the second study was to further investigate the specific details of the intervention, in particular, to explore in more detail the use of NRT for temporary abstinence in the home to protect children from ETS. The interview guides were developed from the literature and through discussions with the Children’s Centre and health visitor staff that provided the authors with useful insights into the social challenges facing their clients. The guides were modified further throughout the data collection to incorporate any new themes. Guide modification was facilitated by immediate post-interview de-briefing between the interviewers. The first set of interviews was carried out by both LLJ and OA and the second set of interviews carried out by LLJ and another interviewer. The discussion guide included exploration of topics around previous quit attempts, previous and future use of NRT, the concept of temporary abstinence and the use of NRT for optional temporary abstinence in the home. We anticipated that the concept of temporary abstinence might be challenging for interviewees to understand and therefore invested time and effort during interviews to help the participants comprehend this. Once we were satisfied that participants adequately understood the concept of temporary abstinence, we explored their views and attitudes towards initiating and maintaining optional temporary abstinence in their homes in general, and also to using NRT for optional temporary abstinence in their homes. The participants were also provided with a NRT summary sheet prior to the interview which included a brief description of some NRT products. For those participants who had no previous experience of NRT, we showed them gum and lozenge samples as visual aids. We have previously analysed the first interview study [15] to show that caregivers had some knowledge of the health risks of ETS exposure for their children, but that they did have some confusion about, and resistance to these health messages; in neither study did we provide caregivers with education about health risks but rather we explored their views and attitudes to temporary abstinence and NRT in the context of their existing knowledge and experience. This study was conducted with reference to and adheres to the RATS guidelines for qualitative research [18]. Ethical approval for the first study was sought and gained from the University of Nottingham Medical School Ethics Committee (ref: A/5/2009) and for the second study a favourable opinion was given by the Nottingham Research Ethics Committee 1 (10/H0403/18).

### Analysis

Data analysis was ongoing throughout the period of data collection and regular discussions were held between the interviewers and within the rest of the research team. Each interview was digitally audio-recorded and transcribed verbatim by an external specialist transcription company. Each transcript was carefully checked for transcription errors to ensure data quality. Analysis of the transcripts was facilitated by the use of NVivo software version 8 (QSR International Pty Ltd, Australia). The interviewers independently generated an open coding framework, utilising the six phase process of Braun and Clarke [19]. The interviewers independently reviewed each transcript and initial ideas were noted that identified preliminary codes. These codes were then grouped into potentially relevant themes and discussed between the researchers and with the wider research team. Further analysis clarified the specific nature of each theme leading to the development of names and descriptions. Following the agreement of the themes identified, extracts were taken from the transcripts to exemplify each theme in order to reflect the experiences of the participants.

## Results

### Participants

In total 36 semi-structured interviews were conducted with self-reported smokers. Although all interviewees were current smokers, they reported a variety of smoking behaviours within the home. Of the 36 interviews, 27 were with mothers, one with a grandmother, a resident carer, and eight with fathers. Eighty one per cent were unemployed or considered themselves to be a house-wife. On average, there were two children living in each household (range, one to six), and in 48% of the households there were two or more adult smokers living in the home.

In this manuscript data relating to the following themes are reported:

1. Participants’ attitudes to the concept of optional temporary abstinence from smoking in the home

2. Participants’ past experiences and perceptions of NRT use

3. Participants’ views about using NRT to help them make their homes smoke-free

### Participants’ attitude to the concept of optional temporary abstinence

Overall, most participants were generally negative about the idea of temporary abstinence in the home. Many did not believe that they could successfully avoid smoking inside their homes (Table 1, quotes a-b). These participants could not recognise the benefit of “just” stopping smoking inside their homes and felt that if they were to invest time and effort to change their smoking behaviour they would prefer to stop smoking altogether (Table 1, quote c). Few caregivers voluntarily raised the subject of their children’s health in this context. One participant, who aspired to quit smoking altogether at some point in the future, did suggest that she would consider making her home smoke-free through optional temporary abstinence, but only as a step towards quitting (Table 1, quote d).

**Table 1:** Participants’ quotes on the concept of optional temporary abstinence

a:	Single male, 25-34: “I don’t think that'd work, smoking outside my home but not in my home d'you know what I mean cos if I've got fags, fags on me whatever d'you know what I mean I'm gonna smoke whether I'm at home or not d'you know what I mean so I'd sooner stop altogether. I don’t think that'd work for me d'you know what I mean not smoking at home… I don’t think I'd stick to not smoking at home if I was smoking outdoor, outside my home d'you know what I mean I think I'd still smoke inside”
b:	Married female, 35-44: “It’s like alcoholics, I, I attribute it to that – alcoholics, if you say to an alcoholic, cos I have an alcoholic in my family, me father. If you say to the alcoholic, we’re not saying you can’t drink, we’re just saying you can drink there – he’ll drink everywhere and I think that would be the same with smoking”
c:	Single female, 25-34: “I think personally, not being funny, I, like I say I don’t think it, I don’t think it’s going to work people do – not, I don’t think people are gonna just do that [abstain] when they’re in the home.. I mean, if you’re going to do it, you’re gonna do it, alright, you’re going to pack it in”
d:	Married female, 25-34: “Possibly to do it in like a two-step phase, to do that one first and then after a while stop altogether…Make the smoke-free house permanent, smoking outside but then, erm, after a while just give up totally”

### Experience with NRT

Most participants reported previous use of NRT and understood that NRT provides nicotine in a medicinal form rather than from cigarettes which helps to overcome the cravings arising from nicotine withdrawal. However, some were still sceptical about the health advantages of using NRT instead of cigarettes (Table 2, quotes a-b). Many of the previous NRT users reported positive experiences and had managed to stop smoking for periods of time, ranging from days to several years. Those who had relapsed rationalised their negative experiences of NRT in terms of the common side-effects and its ineffectiveness. For instance, some users attributed their failed quit attempts to experiencing persistent cigarette cravings despite using high strength NRT products; others were sceptical about the role of NRT as they felt that their own willpower was the most important factor. The taste of NRT was also a common problem – mostly reported for gum, but also experienced with the lozenge and inhalators (Table 2, quotes c-d).

**Table 2:** Participants’ quotes on the previous experience with nicotine replacement therapy

a:	Cohabiting female, 25-34: “I kind of understand it…but then on the other side of it I think because it's nicotine replacement so how is it gunna help you stop if it's still giving you the nicotine”
b:	Single female, 16-24: “You're still getting your erm like your dose of nicotine through whatever you're doing so what's the point in doing it?”
c:	Single female, 16-24: “Like I say I think it's a waste of time. It's all about willpower …”
d:	Married female, 25-34: “I had the gum years ago…but erm and it tastes horrible, it tastes like erm, I know when people say it's not like that now but it used to taste like you were chewing an ashtray”

### Use of NRT for optional temporary abstinence in the homes

Exploration of interviewees’ previous experiences of NRT often facilitated lengthy discussions about the potential use of NRT to help the participants make their homes smoke-free. An overwhelming majority of interviewees expressed generally negative attitudes about using NRT to help them to avoid smoking in their homes. One of the participants, a father of one, voluntarily described how he had already managed to initiate and maintain temporary abstinence to make his home completely smoke free by using nicotine gum to control his cravings inside the house, even though he was not familiar with the terminology of temporary abstinence. His reason for stopping smoking inside the house was his son’s health. However, when asked about the potential use of NRT by other smokers to help them stop smoking in the home, he promptly talked about his desire to use NRT to help him stop smoking altogether (Table 3, quotes a-c). Some participants rejected the use of NRT for optional temporary abstinence because they perceived that stopping completely was the only appropriate way to alter their smoking behaviour, reiterating previously expressed negativity about the concept of temporary abstinence itself (Table 4, quotes a-c). Those participants who had already successfully managed to make their homes smoke-free were also unsure about the value of NRT. These families, as a whole household, appeared to prioritise their children’s health over smoking indoors. They felt that understanding the harmful effects of cigarette smoke in the home was a more important factor. For them, access to NRT alone would not be sufficient to motivate families to stop smoking in the home. They suggested that NRT use had to be supplemented by parental education around the benefits of a smoke-free home (Table 4, quotes d-e).

**Table 3:** Quotes from a participant who successfully used NRT to achieve temporary abstinence in his home

a:	Cohabiting male, 16-24: “Just thought it would be easier instead of smoking inside, it’s easier just to go outside and then your son’s safe and your kids are safe then if you don’t smoke in front of them”
b:	“That’s when [I had cravings] I used to chew the, er, chewing gum as well. A couple of hours it used to work then I’d just think, used to think go outside and have one”
c:	“I’d love to start using like stuff like that to quit altogether as well”

**Table 4:** Participants’ quotes on the use of NRT for optional temporary abstinence in the homes

a:	Married male, 35-44: “No, I think for me I have to stop and stop straightaway in one go, stop and that would be it and then never smoke again, be like an alcoholic that doesn’t drink anymore. I’d still be a smoker that doesn’t smoke”
b:	Cohabiting female, 25-34: “Personally I'd like to in the long run I would like to be smoke-free altogether”
c:	Single female, 16-24: “Well, I wouldn’t see much point in that [using NRT for TA] to be honest if I was, if I was going to stop smoking, if I was going to use something like that [NRT] I’d want to stop smoking completely, not just in the house. You know, because that way I wouldn’t be cheating going outside for a cigarette or – I really do need to stop smoking myself, for myself, for the children cos that way I’m going to be here longer hopefully”
d:	Married female, 35-44: “I would say gums and patches and er I dunno, what else, some kind of maybe not knowledge but I would need to make my own mind right this is the moment, we are not smoking at home. That’s all, end of the story. It would harm our er children so we are not smoking at home any more. If I can't say right maybe I will get the chewing gum or patches but I think you need to get this kind of understanding how, how I'm around children, this is not the place to, to smoke”
e:	Single female, 25-34: “I don’t know, I just can’t see it [use of NRT] cos it might, you don’t know why, people don’t know the effects, it’s going to make you feel odd… I think they’ve got to be put, put more out the reasons why people want it, to make the house as smoke-free. They want to put it out more to people why they want to make, they want the house smoke-free”
f:	Cohabiting male, 16-24: “I tried it once, none of 'em worked so there's no point”
g:	Single female, 16-24: “I just don’t think it works. I think it's more willpower… And if you, if you’ve got the willpower you’ll say no, I'm no, I'm not going to. See I haven’t and I’ll say oh I want a fag anyway. So I just think it's a waste, in my mind I think it's a waste of time”
h:	Married female, 35-44: “I think a lot of it is about you wanting to at that time and the will power and you can sit and you can say, oh I've had these patches on for 2 weeks and not done anything but if you're wearing the patches and still constantly doing your same habit with your cigarettes, I mean, you're not going to be able to give up. I think it's got a lot to do like, if I want to give up and I take everything, you know, and I try every single thing but then at the same time I still keep my cigarettes in my everyday routine I'm not gunna give up am ?”
i:	Single female, 25-34: “I don’t know how much of sort of my anxiety with it is about, I s'pose m’ obviously it's a lot to do with the nicotine but also the sort of give myself five minutes space or fresh air or out on your own sort of thing so I don’t know how, cos I've never really tried them I don’t know how much my sort of anxiety’s to do with just wanting to sort of get out and have, do that ritual or if it's actually just the nicotine sort of thing yeah”
j:	Cohabiting male, 16-24: “I’d love to use it [NRT] to cut down and stuff like that. I don’t really want to smoke for the rest of my life either really… I’d probably try and cut down first then try and quit altogether”
k:	Single female, 25-34: “The idea of the thought of just absolutely stopping is quite like terrifying but I think your thought when you're always thinking I can have one in a bit sort of thing and then use something to sort of take the edge off your sort of cravings I think yeah that would be really helpful yeah, yeah…I s'pose cut down and then, and then, get to a point where you feel you're able to quit”
l:	Single female, 35-44: “I'd erm go on the patches … and the inhaler. So I've got something in my hand … Then I'd cut down slowly as much as I could”
m:	Single male, 25-34: “Oh definitely I'd have patch, gum or lozenges … definitely if that were the case where I had my daughter full time d'you know what I mean definitely on spot… Yeah stop completely d'you know what I mean. I don’t I, d'you know what I mean I'd sooner just stop… I'd sooner not smoke altogether d'you know what I mean I don’t see the point and that d'you know what I mean just having a smoke, but then smoking outside d'you know what I mean I'd sooner not smoke altogether”

Others were sceptical that using NRT for optional temporary abstinence alone would not address the more important need to change their smoking routine (Table 4, quotes f-h). One of the participants, a single mother, felt that smoking for her was more than just nicotine. She was therefore worried that by substituting cigarettes for NRT she would miss out on the opportunities to have a break and time to herself (Table 4, quote i). A small group of participants, when asked directly, were initially positive about using NRT for optional temporary abstinence, however, none gave detailed suggestions as to why this might be and most only briefly indicated agreement. Those few participants who gave explanations talked about using NRT in the context of quitting or cutting down smoking, rather than optional temporary abstinence. The majority of the participants who rejected the idea of using NRT for optional temporary abstinence, however, reported being very willing to use NRT in more established and traditional ways, for example, for quitting or cutting down to quit (Table 4, quotes j-m). This was despite their previous failed attempts and reported side-effects of NRT.

## Discussion

To our knowledge, this is the first study reporting smokers’ views on the value of trying to achieve temporary abstinence from smoking when in the home and the potential role of NRT in this context. We found a generally negative attitude both to the concept of optional temporary abstinence and the use of NRT to achieve it, for a number of reasons. First, many smokers felt that the strength of their addiction and their deeply ingrained smoking routines would be too difficult to overcome for them to stop smoking temporarily whilst inside their home. A number of caregivers also stated a preference for trying to stop smoking altogether, rather than “just” stopping in the house. Despite the fact that most of our participants had previously made failed quit attempts, many would still consider using NRT to help them to quit, either abruptly or after cutting down.

In our previous analysis, we showed that interviewees had an incomplete knowledge and confusion about, and a lack of engagement with the effects of ETS on children’s health [15] and this may have influenced their lack of enthusiasm for optional temporary abstinence. A UK study from 2003 found that 86% of parents who smoked were aware that ETS was harmful to their children’s health [6]. Nevertheless, many parents from the same study were using measures to protect their children from ETS which have been shown to be ineffective, suggesting that many parents underestimate the risks of ETS. Mothers’ perceptions of children’s health risks from ETS has been explored in more detail in other studies, which found that they tended to rely on alternative explanations for ill health unrelated to smoking and falsely viewed older children as more resilient to the effects of ETS [12,13]. In our study, few caregivers who smoked in the home appeared to link our discourse about temporary abstinence to their children’s health. Instead, the emphasis amongst our participants seemed to be around personal goals, such as cutting down or the desire to stop smoking completely, rather than temporarily abstaining from smoking in their homes to benefit their children. Conversely, however, interviewees who did not smoke in their homes believed that knowledge about the harmful effect of ETS on children’s health and prioritization of children’s health were the key issues to making a transition to smoke-free homes. This supports the findings from a Swedish study [20] which showed that parental level of evidence based knowledge regarding the potential risks of ETS to young children might have significantly affected their willingness to protect their children from ETS. We did not attempt to educate parents about the health risks of ETS and it is possible that our results would have been different if we had done so. However, collectively, our findings suggest that providing better knowledge and understanding of these risks would be an essential part of any future intervention to promote smoke-free homes.

It is also possible that the failure to engage with the concept of temporary abstinence in our study group could be partly explained by the relative lack of exposure to situations where the smokers have to abstain from smoking. Many of our participants were unemployed or homemakers, and it is likely that they spend less time in situations of enforced temporary abstinence from smoking than more advantaged households with higher employment rates where smokers are potentially more used to the concept of enforced temporary abstinence and could then transfer this approach to their homes. A major strength of our study is that we explored the views of socio-economically disadvantaged smokers whose children would, potentially, have the most to gain from reduced exposure to ETS. However, it is not clear how closely the findings from this sample would relate to more affluent households where children have less exposure to ETS in the home [11].

Most of our participants had previously tried unsuccessfully to stop smoking, and therefore may have had more negatively biased views about the use of NRT, which were described in other studies [21-23]. We found that some resistance to optional temporary abstinence and NRT came from the complex role of smoking in the lives of these disadvantaged families. Nicotine addiction *per se* appeared to play a small part compared to the much-valued need to have a break and “me” time, which smoking afforded, a finding that is in agreement with previous studies [13,14]. It was almost seen as “cheating” or cutting corners to use NRT if the smokers were not determined to radically change their lifestyle. This belief emphasizes the major effort required of these families to change their smoking behaviour in the home, as reported elsewhere [24].

Despite the negative views, there was still some support for using NRT to achieve optional temporary abstinence, as demonstrated by a father who used NRT successfully to stop smoking in the home and another participant who could see a role for NRT and optional temporary abstinence as a first step towards quitting. Of note, most of our failed quitters could still see themselves using NRT in more traditional ways, in particular, for smoking reduction – either through cutting down, or cutting down to quit. With appropriate support to ensure smokers cut out the cigarettes smoked in the home, such smoking reduction could lead to benefits to children, as well as the smokers’ personal health. A possible implication for healthcare professionals who are involved with smoking families is that it is important to take a person-centred approach, exploring smoker’s previous experiences and knowledge about temporary abstinence and NRT and use them as positive levers to make a permanent change in their household smoking habits.

The findings of our study provide insight into the relatively unexplored ways of protecting children from ETS, which could inform any future interventions around smoke-free homes and ETS exposure. Future research around clinical interventions could explore the possible effectiveness of education on the risks of ETS and lack of effect of partial restrictions of smoking in the homes.

## Conclusions

Caregivers in our study did not engage with the concept of temporary abstinence as a means of protecting children from ETS at home. This could be partly explained by the complex social and psychological aspects of smoking in the lives of these disadvantaged families but also by a lack of knowledge about the specific health risks to children of ETS exposure. We also identified a lack of support for using NRT to achieve temporary abstinence in the home, which may be attributed to people’s incorrect beliefs about the efficacy of NRT. Nevertheless, our study suggests that there is still a role for NRT as some families may be receptive to the idea of using NRT for optional temporary abstinence, and others might be open to using NRT to help them cut down, or cut down to quit and could be encouraged to do so in a way to protect their children’s health. These conclusions, however, may only be valid for disadvantaged families.

## Abbreviations

ETS: Environmental tobacco smoke; NRT: Nicotine replacement therapy

## Competing interest

The authors declare that they have no competing interests.

## Authors’ contributions

OA drafted the manuscript, contributed to the development of the interview guide, carried out interviews and lead the analysis and interpretation of results. TC, AM and SL were involved in the design of the study and the interview guide, participated in discussions around interpretation of the results, commented on drafts of the manuscript and critically reviewed the final version. LLJ contributed to the study design and interview guide development, carried out interviews, was involved in analysis and interpretation of results, commented on drafts of the manuscript and critically reviewed the final version. All authors read and approved the final manuscript.

## Pre-publication history

The pre-publication history for this paper can be accessed here:

http://www.biomedcentral.com/1471-2458/13/262/prepub
